# Pulmonary Enteric Adenocarcinoma in a Young Man

**DOI:** 10.5152/eurasianjmed.2023.22273

**Published:** 2023-02-01

**Authors:** Sevilay Özmen, Funda Demirağ, Fatih Alper, Onur Ceylan, Betül Gündoğdu, Ömer Araz, Çiğdem Kahraman, Adem Maman, Metin Akgün

**Affiliations:** 1 Department of Pathology, Atatürk University, Faculty of Medicine, Erzurum, Turkey; 2 Department of Pathology, Atatürk Chest Diseases and Thoracic Surgery Training and Research Hospital, Ankara, Turkey; 3 Department of Radiology, Atatürk University, Faculty of Medicine, Erzurum, Turkey; 4 Department of Pulmonary Disease, Atatürk University, Faculty of Medicine, Erzurum, Turkey; 5 Department of Medical Genetics, Atatürk University, Faculty of Medicine, Erzurum, Turkey; 6 Department of Nuclear Medicine, Atatürk University, Faculty of Medicine, Erzurum, Turkey

Primary pulmonary enteric adenocarcinoma (PEAC) is an extremely rare subtype of non-small cell lung cancer. Diagnosis requires exclusion of primary enteric carcinoma by gastroscopy and enteroscopy. Primary PEAC is characterized by pathological features similar to colorectal adeno carcinoma and is defined as pulmonary adenocarcinoma. It is an adenocarcinoma with an enteric differentiation component exceeding 50%.^1^ Most patients diagnosed with PEAC usually of advanced age. Our case is the fourth young patient mentioned in the literature.^2-4^

A 24-year-old male was admitted to the chest disease clinic of our hospital with complaints of shortness of breath, cough, and sputum. On thorax computed tomography (CT), a 2 × 3 cm mass is observed in the right parenchyma and hilar region. In Positron emission tomography (PET)-CT scan, the 2-Deoxy-2-fluoro-D-glucose (FDG) affinity of the mass in the thorax is evaluated as low and there are no hypermetabolic foci that can be evaluated as pathological in the gastrointestinal tract and abdomen. A tru-cut biopsy is performed from the patient's right axillary lymph node. Histopathological examination of lymph node material reveals neoplasia in which atypical cells with hyperchromatic nuclei and large eosinophilic cytoplasm form glandular structures (Figure 1). Gastrointestinal system malignancies were excluded endoscopically and colonoscopically. Endobronchial ultrasonography (EBUS) is then performed from the mediastinum for clinical staging. Here, too, adenocarcinoma was observed in which signet ring cells and sometimes high columnar and cuboidal cells were formed by nuclear pseudostratification. In the immunohistochemical study, positive reactivity was observed with Cytokeratin 7 (CK7), Cytokeratin 20 (CK20), and caudal type homeobox 2 (CDX2) in neoplastic cells, while thyroid transcription factor-1 (TTF-1) was evaluated as focal reactive. No pathogenic variant was detected in gene mutation analysis. In the case where malignancy was excluded on gastrointestinal system (GIS), the findings were evaluated as compatible with “Pulmonary enteric adenocarcinoma.”

Since morphological features alone are not sufficient for the diagnosis of PEAC, immunohistochemical markers and gene mutation profiles are important for pathological diagnosis.^3^ Our case is important because it is the fourth youngest patient in the literature. In Cai-Xia Wang et al's^4^ series of 9 cases diagnosed with PEAC, only 1 case had positive expression of all 4 colonic markers (CK20, CDX2, Mucin 2 (MUC2), and villin). In the literature, CK7 and CDX-2 are immunohistochemical markers that are mostly expressed in PEACs.^3^ In our case, immunohistochemically positive, CK-7, CK-20, CD-X2, and TTF-1 were weakly positive focally and similar to the literature. KRAS mutation is common in cases.^2,5^ No pathogenic variant was found in the gene mutation analysis of our case. Due to the rarity of the disease, the prognosis of PEAC patients could not be determined. In the studies in the literature, the 5-year survival rate is very low in most of the cases.^4^ In our case, death occurred in the second year, which is consistent with the literature. In conclusion, due to the low incidence, larger case series are needed to well document the clinical and pathological features of this rare tumor.

## Figures and Tables

**Figure 1.**
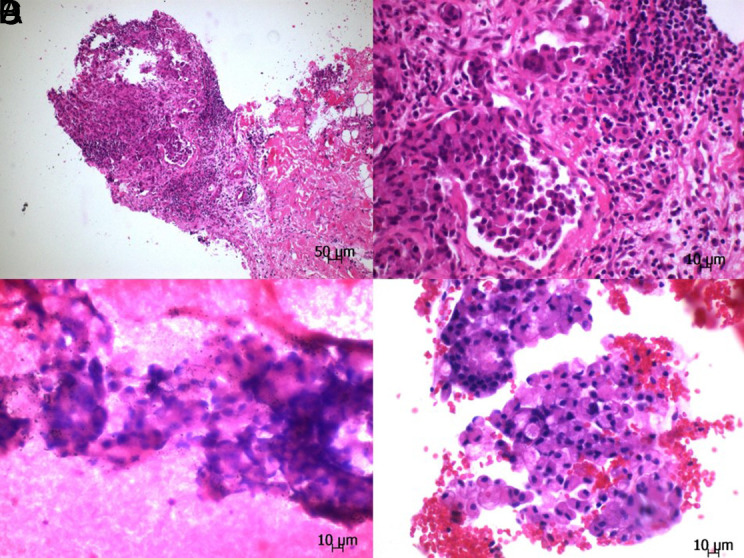
Adenocarcinoma formed by atypical cells infiltrating the lymph node in H&E sections of right axillary lymph node on tru-cut biopsy material (A,B). Adenocarcinoma in which signet ring cells and high columnar and cuboidal cells are formed by nuclear pseudostratification in EBUS material (H&E) (C,D).
